# Understanding Engagement With Digital Mental Health Technology in Mental Health Services: Multicenter Observational Study

**DOI:** 10.2196/67597

**Published:** 2025-08-26

**Authors:** Luke J Borgnolo, Sarah McKenna, Ian B Hickie, Mathew Varidel, Ashlee Turner, Carla Gorban, Haley M LaMonica, Min K Chong, William Capon, Gina Dimitropoulos, Robert Battisti, Bradley Whitwell, Blake Hamilton, Elizabeth M Scott, Frank Iorfino

**Affiliations:** 1 The Brain and Mind Centre, Faculty of Medicine and Health University of Sydney Sydney Australia; 2 Faculty of Social Work, University of Calgary Calgary, AB Canada; 3 Mind Plasticity Sydney Australia; 4 headspace Camperdown Sydney Australia

**Keywords:** digital mental health technology, measurement-based care, mental health services, implementation science, user engagement

## Abstract

**Background:**

Digital technologies can substantially improve mental health care by facilitating measurement-based care through routine outcome monitoring. However, their effectiveness is constrained by the extent to which these technologies are used by services, clinicians, and clients.

**Objective:**

This study aims to investigate engagement with the Innowell platform, a measurement-based digital mental health technology (DMHT), to gain insights into the individual- and service-level factors influencing engagement.

**Methods:**

Participants were 2682 help-seeking clients from 12 Australian mental health services (11 headspace centers and 1 private practice, Mind Plasticity), wherein the Innowell platform was implemented. Although the initial implementation was standardized, services varied in their practical and continued use of the platform, as well as in the resources allocated to foster engagement. All participants completed an initial assessment during onboarding. Engagement was defined as their ensuing completion of the summary questionnaire, designed for routine outcome monitoring. Participants were classified as “initial assessment only,” “single use” (1 completion of the summary questionnaire), or “≥2 uses” (≥2 completions). We analyzed engagement differences across services and associations between engagement and initial assessment scores.

**Results:**

Of the 2682 help-seeking clients, 75.43% (n=2023) completed the initial assessment only, 11.56% (n=310) had 1 completion of the summary questionnaire, and 13.01% (n=349) had 2 or more completions. The service center was the strongest predictor of engagement, with Mind Plasticity participants showing >8 times higher engagement than other centers. At the individual level, higher scores in depression (*P*=.002), mania-like experiences (*P*=.047), suicide ideation (*P*=.004), hospitalization history for mental illness (*P*=.01), and physical activity (*P*<.001) were associated with increased engagement. In contrast, higher levels of anxiety symptoms (*P*=.01), alcohol use (*P*<.001), self-reported mental illness severity (*P*=.02), and social support (*P*=.047) predicted lower engagement. Age and several other clinical variables were not significant predictors when controlling for service-level factors.

**Conclusions:**

This study reveals that both individual- and service-level factors significantly influence DMHT engagement, with the service center being the strongest predictor. This highlights the importance of service-level technology integration and support roles, such as digital navigators, in fostering engagement. Significant variation in engagement among user groups indicates the need for a nuanced approach to measurement-based care. While mental illness generally did not impede engagement, self-perceived severity and anxiety symptoms were barriers. These findings underscore the critical importance of systemic factors and service-level integration strategies in driving DMHT engagement. User-centered designs remain important, but effective integration of DMHTs into existing mental health services is paramount for improving engagement across diverse user groups and clinical presentations. This multilevel approach, encompassing individual, service, and system-wide considerations, is essential for realizing DMHTs’ full potential in delivering effective measurement-based care.

## Introduction

### Background

Advancements in digital technologies have transformed the way mental health care can be delivered and accessed. Innovations such as mobile apps, telemedicine platforms, and web-based therapy have widened the availability of mental health resources and services [1], making support more convenient and readily available to individuals, particularly for those in remote regions or those reluctant to otherwise seek care [2]. Randomized controlled trials have demonstrated the efficacy of digital mental health technologies (DMHTs) [1,3,4], yet their translation into real-world settings remains problematic [5,6]. While randomized controlled trials offer valuable insights into their effectiveness under standardized and prescriptive conditions, real-world implementation is complicated by factors not fully accounted for in such studies. One of these factors is user engagement, shown to be generally lower in real-world settings compared to those in controlled studies [7-11].

Engagement with DMHTs is influenced by individual-related, technology-related, intervention-related, and practical factors [12-14]. However, much of the existing research focuses on stand-alone digital interventions, such as smartphone apps [15], or websites offering mental health resources, activities, or support [16]. The dynamics of engagement with digital technology when used to augment conventional mental health treatment (eg, in-person therapy sessions) remains less understood. Beyond individual factors, addressing this question requires 2 additional factors to be considered: the systemic influences of mental health services and the clinician’s role in integrating the technology into treatment.

The Digital Cumulative Complexity Model (DiCuCoM) [17] provides a useful framework for understanding engagement dynamics applied to different contexts. It articulates a person-centered model of engagement, positing that engagement is driven by the interplay between a patient’s workload (eg, life demands and treatment burden) and their capacity to meet those demands (the sum total of resources and abilities that a patient can draw on to access care, use care, and enact self-care). The model asserts that when workload exceeds capacity, subjective treatment burden increases, leading to lower engagement and poorer outcomes. Consequently, systematically reducing patient workload should be a key priority for improving engagement with digital mental health tools that complement face-to-face treatment.

Measurement-based care (MBC) is a practice whereby clinical decisions are informed by client data collected throughout treatment [18]. This approach supports better-informed and highly personalized clinical decisions [19], with reviews showing that continued monitoring of progress through MBC can reduce deterioration, facilitate dynamic and responsive changes to treatment plans, and enhance treatment effects [20-24]. However, fewer than 20% of mental health providers use MBC in their practice [25], with uptake being hindered by barriers at the level of the client, the clinician, and the organization [26,27]. Consistent measurement is a necessary precondition for the emergence and effectiveness of MBC in treatment and health systems. While digital technologies offer potential solutions to scale MBC implementation, this requires clinicians and services to integrate the DMHT into their care routines and clients to regularly complete their assessments. While MBC has demonstrated positive outcomes in mental health treatment, its effectiveness is constrained by the extent to which both clients and clinicians engage with MBC platforms.

Given these engagement challenges, DMHTs that can seamlessly integrate into clinical workflows while reducing user burden are particularly valuable. Innowell is a measurement-based DMHT designed to support personalized MBC in mental health settings [28]. Three key features make Innowell particularly suitable for investigating multilevel engagement factors: (1) unlike stand-alone interventions, it was developed as an adjunctive tool integrated with face-to-face clinical care; (2) its implementation across multiple centers with identical technology creates a natural experiment to examine nontechnological engagement factors; and (3) its comprehensive multidimensional assessment framework addresses core domains relevant to mental health outcomes. The platform’s multidimensional framework assesses 5 areas: social and occupational functioning; suicidal thoughts and behaviors (STBs); substance misuse; physical health; and illness type, stage, and trajectory.

Across Australia, Innowell has been implemented in 12 primary mental health care centers, with most of these being headspace centers, the nation’s established youth mental health initiative [29]. These centers focus on offering early intervention for mental health problems in young people aged 12 to 25 years [25]. Following initial assessment at intake, routine outcome monitoring is administered via a shorter summary questionnaire, facilitating the capture of a comprehensive clinical picture to support prevention, early intervention, treatment, and continuous monitoring of mental ill health and the maintenance of well-being [19,28].

### This Study

This study aims to enhance understanding of the factors that contribute to user engagement with the Innowell platform, exploring both the systemic and individual factors that influence engagement. Because the technology is identical across the 12 services where it is used, this investigation will discern the differences in engagement between the services and attribute these differences to system- and individual-related factors. Given that the platform is designed to be used as an adjunct to face-to-face treatment, we posit that the implementation of the platform with the service and its integration (by the clinician) into treatment will greatly influence participant engagement with the platform and continued routine outcome monitoring. In addition, we seek to build upon the existing engagement literature by examining the clinical, demographic, and behavioral characteristics of participants at the time of their onboarding, aiming to identify how these factors may affect their subsequent engagement.

## Methods

### Ethical Considerations

This study was approved by Northern Sydney Local Health District Human Research Ethics Committees (HREC/17/HAWKE/480), and all participants provided web-based informed consent (via an opt-out process) [30]. Parental written informed consent was required for those aged <14 years in accordance with Australian laws [30], and participant consent was also obtained. All data collected was deidentified before analysis to ensure participant confidentiality, with personal identifiers replaced by unique codes. Participants did not receive any financial compensation for their participation in the study.

### Participants

Participants were help-seeking individuals who presented for the first time to 1 of 12 mental health services. All clients who presented to these services were invited to use the Innowell platform as part of standard clinical care. During the onboarding process to Innowell, clients were asked if they would consent to having their deidentified data included in research studies. Only those who provided this consent were included in this study, with no additional selection criteria applied. In total, 11 of the 12 services were headspace centers, from urban and regional areas of Australia. headspace centers are publicly funded services focused on providing early intervention mental health support for young people aged 12 to 25 years. The remaining participating service was Mind Plasticity, a private, specialist practice in Sydney, which offers multidisciplinary care to individuals of all ages who require mental health support including psychology, psychiatry, occupational therapy, and neuropsychology, among other health services.

### The Innowell Platform Measures

The Innowell platform is a measurement-based DMHT that aims to assist the assessment, management, and monitoring of mental ill-health and promote the maintenance of well-being. It was part of standard clinical care for individuals presenting to these centers to be directed to the Innowell platform. After being sent an invitation to join the platform, participants completed the initial assessment before their face-to-face appointment with a clinician.

The initial clinical questionnaire assesses various mental health concerns, comorbid and associated risk factors, demographics, physical and mental health history, and prior treatment-seeking behaviors. These questions are from evidence-based screening and assessment tools. Further details about the included assessments can be found in previous publications [19,28,30,31]. After the initial questionnaire was completed, participants could complete a short summary questionnaire or complete measures relevant to specific health domains. Completion of any of these measures could either be recommended by their clinician or be completed voluntarily. The summary questionnaire is a shorter assessment designed to be used repeatedly as an ongoing part of treatment and assessment plans to track key outcomes related to STBs, social and occupational functioning, mental illness severity, social support, and overall health during the treatment period.

There are also options to complete assessments relevant to specific health or mental health domains, known as health cards. These domains include substance misuse, anxiety, depressed mood, eating behaviors and body image issues, mania-like experiences, physical health, posttraumatic stress, psychological distress, psychosis-like experiences, overall health, self-harm, sleep-wake cycle disruption, social and occupational function, social connectedness, and STBs. The summary and health assessments contain a mix of standardized and validated measures, or adapted versions of these measures.

### Implementation of Innowell Across Centers

The implementation and use of the Innowell platform varied significantly across the different centers. This was highlighted in McKenna et al [10], who investigated the implementation and use of the Innowell platform at 2 centers, Mind Plasticity and headspace Camperdown. Although a standardized phased approach to implementation was followed—comprising (1) scoping and feasibility, (2) co-design and preimplementation, (3) implementation, and (4) sustainability and scalability [32]—the integration of the platform into standard care after initial implementation was left to the discretion of each service [33]. For instance, headspace Camperdown mainly used the platform for its initial questionnaire, using it to onboard new patients before their first face-to-face appointment with a clinician. In contrast, Mind Plasticity offered Innowell’s initial and summary questionnaires to their existing clients of the practice, primarily for the purpose of routine outcome monitoring. In addition, Mind Plasticity also integrated a digital navigator as part of the EMPOWERED clinical trial [34]. The digital navigator was a person with lived experience who assisted the service in establishing the system processes and best practices around using Innowell, provided ongoing support to clients and clinicians, and provided services to enhance engagement with Innowell and improve overall service integration [35]. One major goal of the digital navigator was to provide support to clinical teams and clients, helping to remove barriers to engagement and support the integration of the technology into treatment by clinicians and services.

### Statistical Analysis

All statistical analyses were performed using R Studio (version 2023.6.1.524). Engagement was operationalized as the number of times participants completed the summary questionnaire beyond their initial assessment. Due to a skewed engagement distribution, participants were classified into three groups based on their engagement with the summary questionnaire as follows: (1) “initial assessment only” (those who onboarded onto Innowell but did not engage with the summary questionnaire, 2023/2682, 75.43% of the sample); (2) “single use” (those who completed the summary questionnaire once, 310/2682, 11.56% of the sample); and (3) “≥2 uses” (those who completed the summary questionnaire ≥2, 349/2682, 13.01% of the sample). This classification was not predetermined but emerged from the observed distribution pattern. Completion of the summary questionnaire was chosen as our metric for engagement, as it aligned with its purpose as a method of routine outcome monitoring in treatment.

For nonbinary data, we used the Kruskal-Wallis test to compare scores on measures in the initial assessment between the groups. For binary data, chi-square tests were used for between-group comparisons. Post hoc pairwise comparisons on significant differences between the different engagement groups were conducted using Dunn tests and pairwise chi-square tests for binary data. The Bonferroni correction method was used to adjust for the family-wise error rate across the 3 comparisons conducted for each variable, setting the significance threshold at *P*<.017.

A multiple regression analysis was used to control for varying demographics and center-specific effects. This analysis assessed the influence of demographic and individual factors on the number of times they completed the summary questionnaire. Due to the high proportion of 0 counts in the data and potential overdispersion in regular Poisson models, a zero-inflated Poisson regression model was selected. This model accommodated the excess zeros and dispersion by combining a Poisson count model with a log link for nonzero counts and a binomial model with a logit link to model excess zeroes [36], providing a more accurate representation of the data distribution.

## Results

### Sample Description

The final sample included 2682 participants who were onboarded onto the Innowell platform when they presented to 1 of the 12 primary mental health care centers. The mean age was 21.70 (SD 7.30) years, and the sample comprised 1758 (65.55%) female individuals. The summary questionnaire was completed on average 1.08 (SD 3.86) times by each participant, with the number of completions ranging from 0 to 53.

Across the 12 centers, headspace Camperdown had the greatest number of participants (n=1036, 38.63%), followed by Mind Plasticity (n=500, 18.64%). Because specific implementation efforts were made at headspace Camperdown [10], this center was separated from the other headspace centers. Differences in engagement with the summary questionnaire across the different centers are shown in Table 1. Significant differences in participant engagement were identified across Mind Plasticity, headspace Camperdown, and the 10 other centers using the Kruskal Wallis test (*H*_2_=385.51, *P*<.001). Pairwise comparisons using Dunn test indicated that Mind Plasticity had significantly higher engagement than headspace Camperdown (*z* score=15.24, *P*<.001) and the combined 10 other headspace centers (*z* score=18.24, *P*<.001). In addition, headspace Camperdown showed significantly higher engagement than the combined 10 other headspace centers (*z* score=3.46, *P*<.001).

**Table 1:** Descriptive statistics for user engagement with the summary questionnaire across various services, detailing the number of completions of the summary questionnaire by users presenting to certain services (N=2682).

Center name	Completions, mean (SD)	Completions, median	Completions, minimum	Completions, maximum	Total completions	Unique users, n (%)
Mind Plasticity	4.19 (7.74)	1	0	53	2093	500 (18.64)
headspace Camperdown	0.48 (1.58)	0	0	22	493	1036 (38.63)
10 other headspace centers	0.26 (1.14)	0	0	31	302	1146 (42.73)

### Comparisons Between Engagement Groups

The 3 groups classified by their engagement with the summary questionnaire (initial assessment only, single use, and ≥2 uses) were analyzed based on their initial self-assessment. Kruskal Wallis tests and chi-square tests (for binary data) showed significant differences between these 3 engagement groups across several domains (age: *H*_2_=70.29, *P*<.001; help-seeking behaviors: *χ*^2^_2_=31.1, *P*<.001; current treatment status: *χ*^2^_2_=135.3, *P*<.001; diagnosed physical health history: *χ*^2^_2_=6.3, *P*=.04; diagnosed mental illness history: *χ*^2^_2_=22.5, *P*<.001; hospitalization history for mental health: *χ*^2^_2_=59.1, *P*<.001; mania-like symptoms: *H*_2_=16.88, *P*<.001; psychological distress: *H*_2_=19.54, *P*<.001; psychosis-like symptoms: *H*_2_=15.52, *P*<.001; alcohol use: *H*_2_=44.60, *P*<.001; tobacco use: *H*_2_=10.69, *P*=.005; cannabis use: *H*_2_=11.04, *P*=.004; disordered eating: *H*_2_=7.46, *P*=.02; self-harm history: *χ*^2^_2_=12.0, *P*=.002; and daily physical activity: *H*_2_=27.80, *P*<.001). Engagement groups did not significantly differ in their initial assessment of mental health severity, suicidal ideation, suicide intention, or overall health rating; not in employment, education, or training status; not in anxiety symptoms, depressive symptoms, or experience of a traumatic event.

### Pairwise Comparisons

Following significant tests, pairwise comparisons between the 3 engagement groups (Table 2) were conducted using either Dunn test or chi-square tests (for binary data). A critical *P* value of .017 was obtained using the Bonferroni method. Dunn tests revealed that the “initial assessment only” group was significantly younger than both the “single use” (*z* score=3.75, *P*<.001) and the “≥2 uses” (*z* score=7.94, *P*<.001) groups.

**Table 2:** Initial assessment scores, group comparisons, and pairwise comparisons between engagement groups (N=2682)^a^.

Measure			Initial assessment only group		Single use group		≥2 uses group		Between-group comparison		No versus single use group		Single use versus ≥2 uses group		No versus ≥2 uses group
Participants, n (%)			2023 (75.43)		310 (11.56)		349 (13.01)		—^b^		—		—		—
Female participants, n/N (% of group)			1326/2023 (65.55)		196/310 (63.23)		236/349 (67.62)		*χ*^2^_2_=11, *P*=.36		—		—		—
Age (y), mean (SD)			20.88 (6.17)		22.72 (7.76)		25.59 (10.80)		*H*_2_=70.29, *P*<.001		*z* score=8.03, *P*<.001		*z* score=–3.50, *P*<.001		*z* score=3.02, *P*=.001
**Clinical characteristics**															
	Mania-like experiences (ASRM-5^c^), mean (SD)	2.99 (2.94)		2.47 (2.89)		2.63 (2.97)		*H*_2_=16.88, *P*<.001		*z* score=–2.72, *P*=.003		*z* score=3.37, *P*<.001		*z* score=0.76, *P*=.22	
	Anxiety (OASIS^d^), mean (SD)	9.24 (4.25)		8.60 (5.15)		8.63 (4.80)		*H*_2_=4.43, *P*=.11		—		—		—	
	Depressive symptoms (QIDS^e^), mean (SD)	13.67 (4.96)		12.47 (6.47)		12.74 (6.10)		*H*_2_=1.37, *P*=.50		—		—		—	
	Disordered eating (EDE^f^), mean (SD)	4.73 (2.93)		4.23 (3.25)		4.47 (3.22)		*H*_2_=7.46, *P*=.02		*z* score=–1.51, *P*=.07		*z* score=2.22, *P*=.01		*z* score=0.70, *P*=.24	
	Self-harm history (B-NSSI-AT^g^)^h^, n/N (% of group)	936/1670 (56.05)		106/202 (52.48)		186/281 (66.19)		*χ*^2^_2_=12.0, *P*=.002		*χ*^2^_1_=0.8, *P*=.37		*χ*^2^_1_=0.5, *P*=.50		*χ*^2^_1_=0.24.43, *P*<.001	
	Psychological distress (K10^i^), mean (SD)	31.35 (8.00)		27.88 (12.34)		28.44 (10.68)		*H*_2_=19.54, *P*<.001		*z* score=–3.90, *P*<.001		*z* score=2.89, *P*=.002		*z* score=–0.51, *P*=.30	
	Psychosis-like experiences, mean (SD)	5.09 (3.84)		4.31 (4.01)		4.66 (3.98)		*H*_2_=5.78, *P*=.06		*z* score=8.03, *P*<.001		*z* score=–3.50, *P*<.001		*z* score=3.02, *P*=.001	
	Experienced traumatic event (PC-PTSD^j^)^h^, n/N (% of group)	447/766 (58.36)		54/105 (51.43)		79/138 (57.25)		*χ*^2^_2_=1.82, *P*=.40		—		—		—	
	Suicide ideation (SIDAS^k^), mean (SD)	8.95 (11.31)		8.21 (10.50)		9.37 (12.13)		*H*_2_=0.403, *P*=.82		—		—		—	
	Suicide intention (CSSRS^l^)^h^, n/N (% of group)	204/1086 (18.78)		27/156 (17.31)		34/188 (18.09)		*χ*^2^_2_=0.23, *P*=.89		—		—		—	
	Severity of mental illness (CGI^m^), mean (SD)	3.52 (1.42)		3.46 (1.44)		3.48 (1.41)		*H*_2_=0.787, *P*=.68		—		—		—	
	Overall health rating (EQ-5DY^n^), mean (SD)	60.14 (24.13)		59.74 (22.93)		57.95 (25.23)		*H*_2_=1.35, *P*=.51		—		—		—	
**Functioning**															
	Work and social functioning, mean (SD)	18.09 (8.57)		17.24 (9.93)		17.37 (9.67)		*H*_2_=0.951, *P*=.62		—		—		—	
	NEET^o^ status^h^, n/N (% of group NEET)	216 (11.1)		25 (9.8)		24 (8)		*χ*^2^_2_=2.8, *P*=.24		—		—		—	
	Social support (SSSS^p^), mean (SD)	7.14 (3.47)		7.34 (3.56)		7.28 (3.23)		*H*_2_=1.75, *P*=.42		—		—		—	
	Social and occupational functioning (SOFAS^q^), mean (SD)	3.39 (1.43)		3.49 (1.48)		3.40 (1.43)		*H*_2_=1.97, *P*=.37		—		—		—	
	Activity level (MET^r^-minutes), mean (SD)	166.47 (174.83)		120.53 (159.14)		164.58 (177.69)		*H*_2_=27.80, *P*<.001		*z* score=–0.49, *P*=.31		*z* score=5.33, *P*<.001		*z* score=3.81, *P*<.001	

**Health history**															
	Previously sought mental health treatment^h^, n/N (% of group)	1261/1744 (72.31)		166/228 (72.81)		247/281 (87.9)		*χ*^2^_2_=31.1, *P*<.001		*χ*^2^_1_=0.01, *P*=.94		*χ*^2^_1_=47.4, *P*<.001		*χ*^2^_1_=347.1, *P*<.001	
	Currently receiving mental health treatment^h^, n/N (% of group)	516/1744 (29.59)		84/228 (36.84)		183/281 (65.12)		*χ*^2^_2_=135.3, *P*<.001		*χ*^2^_1_=4.7, *P*=.03		*χ*^2^_1_=15.8, *P*<.001		*χ*^2^_1_=290.68, *P*<.001	
	Diagnosed mental illness history^h^, n/N (% of group)	1245/1744 (71.39)		170/227 (74.89)		239/282 (84.75)		*χ*^2^_2_=22.5, *P*<.001		*χ*^2^_1_=1.0, *P*=.31		*χ*^2^_1_=56.2, *P*<.001		*χ*^2^_1_=319.1, *P*<.001	
	Diagnosed physical illness history^h^, n/N (% of group)	583/1743 (33.45)		83/228 (36.4)		115/281 (40.93)		*χ*^2^_2_=6.3, *P*=.04		*χ*^2^_1_=0.7, *P*=.42		*χ*^2^_1_=16.9, *P*<.001		*χ*^2^_1_=191.9, *P*<.001	
	Hospitalization history^h^, n/N (% of group)	245/1744 (14.05)		34/227 (15)		91/282 (32.23)		*χ*^2^_2_=59.1, *P*<.001		*χ*^2^_1_=0.1, *P*=.78		*χ*^2^_1_=113.4, *P*<.001		*χ*^2^_1_=901.7, *P*<.001	
**Alcohol and substance misuse**															
	Alcohol use (AUDIT-C^s^), mean (SD)	4.48 (4.03)		3.31 (3.87)		3.27 (3.52)		*H*_2_=44.60, *P*<.001		*z* score=–4.96, *P*<.001		*z* score=5.08, *P*<.001		*z* score=0.52, *P*=.30	
	Cannabis use (ASSIST^t^), mean (SD)	1.69 (2.99)		1.47 (2.76)		1.13 (2.42)		*H*_2_=11.04, *P*=.004		*z* score=–3.41, *P*<.001		*z* score=1.27, *P*=.10		*z* score=–1.42, *P*=.08	
	Tobacco use (ASSIST), mean (SD)	1.54 (2.24)		1.51 (2.26)		1.05 (1.93)		*H*_2_=10.68, *P*=.005		*z* score=–3.38, *P*<.001		*z* score=0.46, *P*=.32		*z* score=–2.04, *P*=.02	

^a^Unless otherwise stated, higher scores indicate higher levels of the measured construct.

^b^Not applicable.

^c^ASRM: Altman Self-Rating Mania Scale.

^d^OASIS: Overall Anxiety Severity and Impairment Scale.

^e^QIDS: Quick Inventory of Depressive Symptomology.

^f^EDE: Eating Disorder Assessment (modified).

^g^B-NSSI-AT: Brief Nonsuicidal Self-Injury Assessment Tool.

^h^Indicates that the assessment is a binary single-item measure, where 1=yes, and 0=no, so scores represent the proportion of the group that responded “yes.” For these binary measures, chi-square tests were used to conduct group and pairwise comparisons.

^i^K10: Kessler Psychological Distress Scale.

^j^PC-PTSD-5: Primary Care Posttraumatic Stress Disorder Screen for Diagnostic and Statistical Manual of Mental Disorders, Fifth Edition.

^k^SIDAS: Suicidal Ideation Attributes Scale.

^l^C-SSRS: Columbia-Suicide Severity Rating Scale.

^m^CGI: Clinical Global Impressions (self-report).

^n^EQ-5D-Y: EuroQol 5-Dimension Youth.

^o^NEET: not in employment, education, or training.

^p^SSSS: Schuster Social Support Scale (higher scores indicate lower social support).

^q^SOFAS: Social and Occupational Functioning Assessment (higher scores indicate higher impairment).

^r^MET: Metabolic Equivalent of Task.

^s^AUDIT-C: Alcohol Use Disorders Identification Test.

^t^ASSIST: Alcohol, Smoking, and Substance Involvement Screening.

Comparing clinical characteristics at their initial assessment, the “initial assessment only” group reported higher mania-like experiences than both the “single use” (*z* score=3.35, *P*=.001) and “≥2 uses” (*z* score=2.83, *P*=.007) groups and higher psychosis-like experiences compared to the “single use” group (*z* score=3.52, *P*<.001). Compared to the “initial assessment only” group, the “≥2 uses” group had higher rates of self-harm history (*χ*^2^_1_=24.4, *P*<.001) but reported lower psychological distress (*z* score=3.78, *P*<.001).

Across the alcohol and substance misuse domain, the “≥2 uses” group reported lower alcohol use (*z* score=5.00, *P*<.001), tobacco use (*z* score=3.27, *P*=.002), and cannabis use (*z* score=3.24, *P*=.002) than the “initial assessment only” group. Furthermore, the “single use” group also reported less alcohol use than the “initial assessment only” group (*z* score=5.08, *P*<.001).

The “single use” group reported lower activity levels (daily metabolic equivalent of task minutes) than both the “initial assessment only” (*z* score=5.26, *P*<.001) and the “≥2 uses” (*z* score=3.84, *P*<.001) groups.

There were significant differences across the health history domain. Those in the “≥2 uses” group had higher rates of previously seeking mental health treatment than the “initial assessment only” (*χ*^2^_1_=347.1, *P*<.001) and “single use” (*χ*^2^_1_=47.4, *P*<.001) groups, more likely to be currently receiving mental health treatment than the “initial assessment only” (*χ*^2^_1_=290.7, *P*<.001) and the “single use” (*χ*^2^_1_=15.8, *P*<.001) groups, had higher rates of previous mental illness diagnosis than the “initial assessment only” (*χ*^2^_1_=319.1, *P*<.001) and the “single use” (*χ*_1_^2^=56.2, *P*<.001) groups, had higher rates of hospitalization history for a mental illness than the “initial assessment only” (*χ*^2^_1_=901.7, *P*<.001) and the “single use” (*χ*_1_^2^=111.4, *P*<.001) groups, and had higher rates of previous physical illness diagnosis than the “initial assessment only” (*χ*^2^_1_=191.0, *P*<.001) and the “single use” (*χ*_1_^2^=16.9, *P*<.001) groups.

### Multiple Regression

While using the engagement groups to analyze the differences in scores at the time of initial assessment revealed some trends, these differences could be influenced by varying demographics across the 12 centers. To control for this potential confound, a multiple regression analysis was performed, including Mind Plasticity, headspace Camperdown, and the combined group of the other headspace centers into the model. The dependent variable was the number of times the summary questionnaire was completed. A zero-inflated model was chosen due to the substantial proportion of 0 counts observed in the dataset, with 75.4% (2023/2682) of the sample reporting no completions of the summary questionnaire [36]. This model comprised 2 components—a count model (Table 3) predicting the count for nonzero observations using a Poisson distribution with log link and a zero-inflation model predicting excess zeros using a binomial distribution with logit link (Table 4). The model’s appropriateness was supported by multiple fit indices. The zero-inflated Poisson model demonstrated a significantly lower Akaike information criterion value of 2935.80 compared to the regular Poisson model with a value of 4228, indicating a better fit to the data. To address potential multicollinearity and maintain model parsimony, the initial set of assessment variables was reduced to 15 based on conceptual overlap and correlation analysis, with age and presenting center additionally included in the regression (Tables 3 and 4). The highest correlation among the retained variables was *r*=0.66.

**Table 3:** Zero-inflated Poisson regression results: count model (N=2682).

	Estimate (SE)	*z* score	*P* value
(Intercept; headspace Camperdown)	1.12 (0.20)	5.59	<.001
Mind Plasticity	1.19 (0.10)	11.77	<.001
Other headspace centers	–0.56 (0.15)	–3.86	<.001
Age	–.01 (0.00)	–1.54	.17
Depressive symptoms	0.03 (0.01)	2.91	.002
Anxiety symptoms	–0.02 (0.01)	–2.82	.011
Mania-like experiences	0.02 (0.01)	1.98	.047
Psychosis-like experiences	–0.02 (0.01)	–1.78	.17
Disordered eating	–0.01 (0.01)	–1.32	.22
Suicidal ideation	0.01 (0.00)	2.97	.004
Suicide intention	0.18 (0.10)	1.91	.06
Mental illness severity	–0.06 (0.03)	–2.25	.02
Receiving current treatment	0.16 (0.099)	1.68	.11
Hospitalization history	0.17 (0.067)	2.49	.01
Social and occupational functioning	–0.06 (0.03)	–2.52	.01
Daily physical activity	0.0007 (0.00)	4.68	<.001
Overall health rating	0.00 (0.00)	–0.52	.60
Alcohol use	–0.05 (0.01)	–6.00	<.001
Social support	0.02 (0.01)	1.99	.047

**Table 4:** Zero-inflated Poisson regression results: zero-inflation model (N=2682).

	Estimate (SE)	*z* score	*P* value
(Intercept; headspace Camperdown)	1.15 (0.62)	1.84	.07
Mind Plasticity	–1.29 (0.27)	–4.77	<.001
Other headspace centers	0.57 (0.21)	2.67	.008
Age (y)	0.00 (0.01)	0.22	.82
Depressive symptoms	0.02 (0.03)	0.54	.59
Anxiety symptoms	–0.03 (0.03)	–1.22	.22
Mania-like experiences	0.05 (0.03)	1.45	.15
Psychosis-like experiences	–0.05 (0.03)	–0.18	.86
Disordered eating	–0.03 (0.03)	–1.02	.31
Suicide intention	–0.12 (0.26)	–0.44	.66
Suicidal ideation	0.08 (0.01)	0.73	.46
Mental illness severity	0.04 (0.08)	0.48	.63
Receiving current treatment	–0.09 (0.02)	–0.44	.66
Hospitalization history	–0.16 (0.215)	–0.75	.46
Social and occupational functioning	0.01 (0.07)	0.15	.88
Daily physical activity	0.001 (0.00)	0.30	.77
Overall health rating	0.00 (0.00)	0.02	.99
Alcohol use	0.04 (0.03)	1.73	.08
Social support	–0.06 (0.03)	–2.13	.03

In the count model (Table 3), several variables were significant predictors of engagement with the summary questionnaire. Participants presenting to Mind Plasticity showed the strongest positive association (β=1.19, *P*<.001), followed by headspace Camperdown (β=1.12, *P*<.001), while “other headspace centers” was negatively associated (β=–0.56, *P*<.001). Other significant positive predictors included suicide ideation (β=0.01, *P*=.004), depressive symptoms (β=0.03, *P*=.002), mania-like experiences (β=0.02, *P*=.047), hospitalization history (β=0.17, *P*=.01), and physical activity (β=0.0007, *P*<.001). Significant negative predictors included alcohol use (β=–0.05, *P*<.001), mental illness severity (β=–0.06, *P*=.02), social and occupational functioning (β=–0.06, *P*=.01), and anxiety symptoms (β=–0.02, *P*=.01). Notably, because the social support measure is reverse scored, with higher scores indicating poorer social support, the positive association (β=0.02, *P*=.047) indicates that participants with lower level of social support showed increased engagement.

In the zero-inflated Poisson model (Table 4), fewer variables were significant. Mind Plasticity was strongly negatively associated with excess zeros (β=–1.29, *P*<.001), while “other headspace centers*”* was positively associated (β=0.57, *P*=.008). Better social support was also associated with excess zeros (β=–0.06, *P*=.03), again reflecting the inverse scoring of this measure. Several variables, including age, suicide intention, psychosis-like experiences, disordered eating, and overall health, were neither significant predictors in the count nor zero-inflation models.

## Discussion

### Principal Findings

This study provides valuable insights into key characteristics of users who engage with a digital technology in mental health services. Specifically, this work shows that there are clear individual differences that separate those who are using the Innowell platform most and least regularly. Importantly, this work underscores the substantial influence of broader structural or systematic factors on the use of DMHTs, with the participants’ treatment center emerging as the strongest predictor of engagement. By providing a detailed and nuanced understanding of DMHT engagement in mental health services, this work has important implications for guiding clinical practice and informing policy decisions aimed at enhancing user engagement.

The Innowell platform is designed to guide and support treatment by a clinician or a service provider and is not intended to be used as a stand-alone tool for medical or health advice, diagnosis, or treatment. Consequently, the utility of the platform for the client is largely dependent on its active integration into treatment by the clinician [35,37,38]. Similar to the systematic barriers to MBC uptake that van Sonsbeek et al [26] describe, this suggests that there exist some obvious service-level factors that influence individual engagement with measurement-based DMHTs. Therefore, independent of the technology itself, the extent to which the platform is integrated into the service and treatment will affect individual engagement in routine outcome monitoring behaviors.

We found that the largest influence of engagement was the service from which participants received treatment. Participants from Mind Plasticity had, on average, >8 times the engagement with the summary questionnaire (mean 4.19, SD 7.74) compared to the next highest-engaging center (mean 0.48, SD 1.58). While the implementation of the Innowell platform across all centers was guided by a strategy for implementation science [32], there were some substantive differences in how different centers embedded the platform as part of standard care [10]. The primary contributing factor was likely that Mind Plasticity used the platform as a means of routine outcome monitoring, whereas other centers primarily used the tool as an initial assessment for patient onboarding. This approach to routine outcome monitoring was enhanced by Mind Plasticity’s implementation of a digital navigator [35], a unique feature among the centers studied.

Digital navigators provided valuable support to clinical teams and clients, helping to remove potential barriers to client engagement and support the integration of DMHTs into treatment by clinicians and services. While traditionally used to support self-guided web-based therapy modules, the increased engagement observed in Mind Plasticity suggests that digital navigators can also be effective in supporting the integration of measurement-based DMHTs into traditional therapies provided by health professionals. The role included providing nonclinical technical support, interpreting data with clients before clinical sessions, and building therapeutic alliances with clients and clinicians around DMHT use [35,39,40]. Crucially, previous findings from Mind Plasticity suggest that the engagement benefits from the digital navigator extend beyond mere client support, that is, clinicians who integrated the digital navigator as part of their care team and maintained frequent contact with them saw higher engagement among their clients. Specifically, following digital navigator contact, clients were more likely to engage with the platform if their health professional had strongly promoted DMHT use and had high engagement with the digital navigator themselves [35]. This underscores the importance of clinician adoption and integration of DMHTs, facilitated by digital navigators, in driving client engagement with DMHTs within traditional therapeutic contexts.

These findings are broadly consistent with the DiCuCoM [17], which posits that engagement is determined by the balance between patient workload and capacity. Our results suggest that structural differences at Mind Plasticity, such as the supportive digital navigator role, can effectively reduce barriers and enhance resources to support higher engagement. However, our study suggests a potential extension to DiCuCoM when applied specifically to adjunctive technologies. While DiCuCoM addresses passive enablers of engagement (reducing workload and enhancing capacity), it insufficiently accounts for active motivational factors crucial for sustained engagement with DMHTs used alongside face-to-face treatment. When technology serves as an adjunct rather than stand-alone intervention, clinician behavior becomes a powerful engagement driver. Clients use platforms such as Innowell primarily when they perceive tangible clinical utility, which is directly signaled through clinician integration of the tool in sessions. Qualitative work within Mind Plasticity showed that when clinicians actively referenced and used DMHT data in treatment planning, they demonstrated concrete value to clients [35,38], creating a motivational framework that extended beyond the workload-capacity balance emphasized in DiCuCoM. Future engagement frameworks for adjunctive technologies should explicitly incorporate these motivational mechanisms that operate at the intersection of clinician behavior and client perception.

The substantial differences in engagement with the Innowell platform between Mind Plasticity and the headspace centers highlight the importance of how a technology is integrated into a service. Mind Plasticity’s high engagement illustrates that the Innowell platform can successfully deliver on its purpose—to facilitate MBC through routine outcome monitoring [30]. Part of their success likely stems from their deliberate integration of the technology into the service; with greater participation from clinicians, use of support roles such as digital navigators, and a greater focus on the delivery of MBC. For any DMHT used as an adjunct to clinical treatment, it follows that integration of the DMHT within the service and the specific provision of support to the clients will facilitate engagement with the technology. The Innowell platform should be seen as a tool whose potential for MBC can only be realized fully when embedded within organizational and clinical frameworks specifically designed to support continuous care and routine outcome monitoring.

Beyond these general implementation factors, it would be naive to suggest that headspace centers could simply adopt Mind Plasticity’s implementation approach (eg, including digital navigator support roles and technology integration in treatment) without addressing fundamental structural differences between private and public mental health service models. Private services, such as Mind Plasticity, typically provide more specialized mental health care, with resources and infrastructure explicitly supporting ongoing treatment and routine outcome monitoring, thereby able to leverage digital tools such as Innowell more effectively. In contrast, headspace centers face inherent structural constraints related to their public funding models, higher clinician workloads, and typically less-specialized care aimed at early intervention and brief treatment [41]. This structural reality is reflected in the 2022 evaluation of the national headspace program by KPMG [42], revealing that a substantial proportion of young people (36%) who accessed a headspace service did so only 1 time, and only 19% had ≥6 occasions of service. Given that MBC assumes multiple visits to monitor progress over time, the observed 75.4% nonengagement rate likely reflects a combination of a service model that emphasizes single-session brief interventions, implementation factors, and a mismatch between clinical care options and the needs of young people presenting for care [43]. While single-session brief intervention approaches may be most appropriate for those presenting with low complexity illness [41], it is estimated that 60% of young people present to headspace centers with more complex needs (ranging from 36% to 79% across centers) [44]. Another empirically driven approach estimated that 48.8% of those presenting to headspace have very high and complex needs, with functional impairment, suicidality, and at-risk mental states, and an additional 27.5% present with an established illness with functional impairment [45]; with individuals fitting into these 2 classifications likely benefiting from ongoing treatment and routine outcome monitoring. Compared to private practices such as Mind Plasticity, headspace services have demonstrated that they are not as well equipped to manage more complex clinical profiles that would benefit from routine outcome monitoring. While DMHTs such as Innowell can be used to help facilitate routine outcome monitoring and MBC, meaningful improvements in engagement, particularly within headspace centers, will require targeted changes beyond simply adopting DMHTs. At a practical level, introducing digital navigators to mitigate technological, logistical, and motivational barriers, alongside targeted clinician training to effectively incorporate platform data into clinical practice, could readily enhance engagement. However, broader structural adjustments are also essential. Given headspace centers’ high prevalence of single-session visits, and the inevitable mismatch between clinical needs and clinical care options that ensues, services should implement mechanisms to better assess clinical complexity at initial visits [44,46], enabling tailored service delivery and consistent routine outcome monitoring (using DMHT) specifically for those clients most likely to benefit from ongoing support.

Despite the strong role of service factors, individual characteristics still significantly influenced engagement. The relationship between clinical presentation at initial assessment and subsequent engagement with the Innowell platform revealed complex patterns. Higher scores in depression, mania, suicidal ideation, and hospitalization history were associated with increased engagement, partially aligning with the findings of Borghouts et al [12] that more severe mental health symptoms generally increase interest in DMHTs. However, our results diverge from their conclusion that depressive symptoms hinder engagement. Interestingly, we found anxiety and self-assessed mental illness severity to be negatively associated with future engagement. This nuanced relationship suggests that DMHT engagement is not simply a function of overall symptom severity but may depend on specific symptom profiles. Factors, such as the perceived value of symptom monitoring, potential avoidance behavior, or varying levels of clinician encouragement, could also contribute to these complex associations. These findings underscore the need for tailored approaches in implementing DMHTs, considering the intricate interplay between clinical presentation and engagement patterns. Importantly, these results demonstrate that, generally, the presence of mental illness is not a barrier to engagement—and can in fact be associated with higher engagement. This insight could inform strategies to optimize DMHT use across diverse clinical groups.

The observed patterns in help-seeking behaviors, physical activity, and substance misuse across engagement groups, and their associations with engagement, could be explained by “health consciousness”—the self-awareness of one’s health and the propensity to pursue health-promoting behaviors [47]. Although not directly measured, engaging with mental health tracking tools, such as the Innowell platform, is a “healthy” behavior and could have contributed to this effect. Help-seeking behaviors and physical activity levels were associated with higher engagement, and substance misuse was associated with lower engagement. Because this pattern aligns with typical “healthy” and “unhealthy” behaviors, it is possible that the construct “health consciousness” could underlie this pattern of differences. Previous literature has shown that comorbid substance misuse is one of the strongest factors associated with noninitiation and nonengagement in mental health treatment [48], and may also act as a barrier to engagement and adherence to treatment [49]. Although speculative and requiring further direct measurement, this idea of health consciousness may offer some explanation for the observed pattern of engagement.

The mean participant age varied significantly between engagement groups; however, these results should be interpreted with caution. The headspace centers serve individuals aged 12 to 25 years, whereas Mind Plasticity accommodates all ages, resulting in a higher average age (29.84, SD 11.6 y) compared to other centers (21.83, SD 3.08 y). This discrepancy, combined with the higher engagement levels from participants from Mind Plasticity, confounds the apparent age-engagement relationship. When age was entered into the regression model, controlling for the presenting center, there was no effect on engagement. Surprisingly, social support was found to be negatively associated with engagement. Previous findings have shown that access to social support via social networks and forums can enhance engagement with stand-alone website- or app-based digital mental health interventions [14,50]. However, in our context, individuals with higher levels of social support may also have more diverse coping strategies and resources available to them, potentially reducing their reliance on digital tools for mental health monitoring. While social support interventions specifically targeted at treatment can be helpful for engagement, already established social support networks may reduce engagement with measurement-based DMHTs. Further research is needed to understand the precise mechanisms underlying this association.

Beyond individual clinical and demographic factors, broader cultural and socioeconomic influences likely shape young people’s engagement with DMHTs and mental health services generally. Within the DiCuCoM framework [17], public mental health services such as headspace may attract individuals facing greater financial constraints, who potentially already have a high “workload” and thus have limited capacity for sustained engagement. In addition, geographic location may influence engagement. For example, regional youth typically have lower rates of previous help-seeking compared to those in urban areas [31,51], and may be more reluctant to engage consistently due to privacy concerns or fear of stigma within small, tight-knit communities. In contrast, inner-city services such as Mind Plasticity might attract individuals already comfortable with seeking mental health support, thereby promoting sustained engagement. Although these factors likely influence sustained engagement with DMHTs, our dataset lacks sufficient granularity to comprehensively explore these sociocultural causes of engagement or disengagement. Future research should explicitly examine these influences to better understand engagement patterns across diverse mental health service contexts.

### Limitations

There are several limitations to be considered. First, the disparity in engagement levels across centers and significantly higher engagement in participants from Mind Plasticity may have introduced some biases. Although this engagement difference can be explained by the center’s emphasis on routine outcome monitoring and platform integration into clinical practice, the center’s location in inner-city Sydney represents a demographic that differs from more regional Australian centers. Though this was controlled for in the multiple regression, previous research has shown that those presenting to urban services were more likely to have previously sought help, and have more problems with alcohol use compared to regional service youth [31]. Although the exclusive focus on Australian services may limit generalizability, the core principle—that effective integration of technology promotes engagement—is likely applicable globally. Future efforts should be made to encourage the adoption of the Innowell platform for routine outcome monitoring purposes across diverse centers to facilitate a broader and more representative analysis of routine outcome monitoring and engagement patterns.

Another limitation of the study arises from the absence of data linking participants to specific clinicians, making us unable to differentiate whether engagement was primarily driven by the individual, the service, or the clinician. Understanding this could foster the development of more targeted approaches to increase engagement. Recent qualitative research has demonstrated the importance of the role of the clinician on engagement with the Innowell platform [35,38]. While maintaining patient and clinician confidentiality, future studies should also track clinician-participant pairings to better understand the clinician’s role in patient engagement.

Finally, engagement is operationalized as completion of the Innowell summary questionnaire, which covers domains of physical health, mental illness severity, functioning, STBs, and social connectedness [19]. The classification into 3 engagement groups (initial assessment only, single use, and ≥2 uses) was not clinically driven, but based on the observed distribution in the data. We addressed this limitation by also using zero-inflated Poisson regression, which modeled engagement as a continuous variable. Findings from both approaches were largely consistent, supporting the robustness of our results across analytic methods. Furthermore, the relevance of the summary questionnaire may vary among individuals. While it broadly assesses mental health and functioning, some participants may have preferred completing repeat measures of specific symptoms or conditions, such as anxiety or depression. Thus, there could be further engagement with the platform not captured by solely looking at the summary questionnaire. However, this definition of engagement is specifically relevant to the context of the Innowell platform, as the summary questionnaire was purpose-built for routine outcome monitoring. Although there have been calls to adopt a standardized definition of engagement, emphasizing the incorporation of multidimensional indices [52], the summary questionnaire, which was designed to track outcomes during treatment, serves as a useful measure of engagement in this context. Nevertheless, future research is needed to define dynamic thresholds for optimal level engagement.

### Conclusions

These findings provide insight into the demographic and clinical factors of users who are more or less likely to interact with DMHTs. Importantly, we also show that the most prominent predictor of participant engagement is the center that they present to. This highlights the role of broader systemic factors in influencing engagement with DMHT. Furthermore, the severity of mental illness at time of initial assessment does not necessarily impede subsequent engagement with the DMHT. We show that the Innowell Platform can be an effective tool for the delivery of MBC. However, the observed variability in engagement across centers suggests effective integration is largely dependent on integration with mental health services, rather than solely on individual user characteristics. Furthermore, our results indicate that low engagement may be partly attributable to a broader systemic issue—the high prevalence of single-occasion visits to mental health services. This highlights a critical challenge in continuity of care that extends beyond DMHT use. These findings underscore the need for a multifaceted approach—developing nuanced, user-centered DMHT designs; promoting comprehensive service-level integration to foster routine outcome monitoring; and addressing systemic issues that lead to discontinuous care. By addressing technological, systemic, and continuity-of-care aspects, DMHTs can become more effective, accessible, and personalized tools to deliver MBC, helping to meet the needs of both users and health care providers while promoting sustained engagement in treatment.

## Abbreviations

DiCuCoM: Digital Cumulative Complexity Model
DMHT: digital mental health technology
MBC: measurement-based care
STB: suicidal thought and behavior

## Data Availability

The datasets analyzed during this study are available from the corresponding author on reasonable request.
